# Potential use of supervised injection services among people who inject drugs in a remote and mid-size Canadian setting

**DOI:** 10.1186/s12889-019-6606-7

**Published:** 2019-03-08

**Authors:** Sanjana Mitra, Beth Rachlis, Bonnie Krysowaty, Zack Marshall, Cynthia Olsen, Sean Rourke, Thomas Kerr

**Affiliations:** 1 British Columbia Centre on Substance Use, 400-1045 Howe Street, Vancouver, BC V6Z 2A9 Canada; 2 Interdisciplinary Studies Graduate Program, University of British Columbia, 270-2357 Main Mall, H.R. MacMillan Building, Vancouver, BC V6T 1Z4 Canada; 3 Institute for Clinical and Evaluative Sciences, 2075 Bayview Avenue, G172, Toronto, ON M4N 3M5 Canada; 4 Division of Clinical Public Health, Dalla Lana School of Public Health, University of Toronto, 155 College Street, 6th floor, Toronto, ON M5T 3M7 Canada; 5 Lakehead Social Planning Council, #38-125 Syndicate Avenue South, Thunder Bay, ON P7E 6H8 Canada; 6 School of Social Work, McGill University, 3506 University Street, Room 300, Montreal, QC H3A 2A7 Canada; 7 Thunder Bay Drug Strategy, City of Thunder Bay, 500 Donald Street East, P.O. Box 800, Thunder Bay, ON P7C 2K4 Canada; 8 Centre for Urban Health Solutions, Li Ka Shing Knowledge Institute of St. Michael’s Hospital, 209 Victoria Street, 3rd Floor, Toronto, ON M5B 1T8 Canada; 9 Department of Psychiatry, University of Toronto, 250 College Street, 8th Floor, Toronto, ON M5T 1R8 Canada; 10 Department of Medicine, University of British Columbia, 608-1081 Burrard Street, Vancouver, BC V6Z 1Y6 Canada

**Keywords:** Supervised injection services, Supervised consumption facilities, Feasibility research, People who inject drugs, Mid-sized cities, Remote communities

## Abstract

**Background:**

While supervised injection services (SIS) feasibility research has been conducted in large urban centres across North America, it is unknown whether these services are acceptable among people who inject drugs (PWID) in remote, mid-size cities. We assessed willingness to use SIS and expected frequency of SIS use among PWID in Thunder Bay, a community in Northwestern, Ontario, Canada, serving people from suburban, rural and remote areas of the region.

**Methods:**

Between June and October 2016, peer research associates administered surveys to PWID. Sociodemographic characteristics, drug use and behavioural patterns associated with willingness to use SIS and expected frequency of SIS use were estimated using bivariable and multivariable logistic regression models. Design preferences and amenities identified as important to provide alongside SIS were assessed descriptively.

**Results:**

Among 200 PWID (median age, IQR: 35, 28–43; 43% female), 137 (69%) reported willingness to use SIS. In multivariable analyses, public injecting was positively associated with willingness to use (Adjusted Odds Ratio (AOR): 4.15; 95% confidence interval (CI): 2.08–8.29). Among those willing to use SIS, 87 (64%) said they would always/usually use SIS, while 48 (36%) said they would sometime/occasionally use SIS. In multivariable analyses, being female (AOR: 2.44; 95% CI: 1.06–5.65) and reporting injecting alone was positively associated with higher expected frequency of use (AOR: 2.59; 95% CI: 1.02–6.58).

**Conclusions:**

Our findings suggest that SIS could play a role in addressing the harms of injection drug use in remote and mid-sized settings particularly for those who inject in public, as well as women and those who inject alone, who report higher expected frequency of SIS use. Design preferences of local PWID, in addition to differences according to gender should be taken into consideration to maximize the uptake of SIS, alongside existing health and social service provisions available to PWID.

## Background

Cities across North America are contending with epidemics of opioid use and fatal drug poisoning linked to injection drug use, as well as other associated harms such as soft tissue infections, endocarditis and blood-borne infections [1–4]. Beyond the individual-level health-related harms experienced by people who inject drugs (PWID), injection drug use in public spaces is perceived as a major community concern, contributing to the improper disposal of syringes and other injection-related materials [5]. Further, costs associated with hospital utilization and emergency room visits related to injection drug use increase the financial burden on health care systems [6, 7].

To address the individual and structural risks associated with injection drug use, supervised injection services (SIS) offer safe and hygienic conditions where people can inject previously obtained illicit substances under medical supervision [8, 9]. The services can also provide clients access to sterile injecting equipment and connections to basic medical care as well as referrals to other health and social services [9]. SIS have been implemented in many settings, including Western Europe and Australia [10]. In Canada, two long-standing SIS have been operating in Vancouver for over a decade [11, 12], with new sites opening in urban centres across British Columbia, Ontario, Alberta and Quebec in the past year [13, 14].

Rigorous evaluations of SIS in Vancouver, Canada and Sydney, Australia have established that SIS have beneficial effects on the communities in which they are situated, improving health and social outcomes associated with injection drug use [5, 15–20]. The services have been attributed to reducing the risk of transmission of blood-borne infections [16, 18] and fatal overdose [17], while increasing the uptake of medical care and addiction treatment [19, 20]. SIS have also been shown to reduce public injecting and improperly disposed syringes and injection-related litter [5, 21], in addition to decreasing the number of overdose-related ambulance attendances in the immediate vicinity of the service [15].

Feasibility research has been used previously to inform the implementation of SIS and establish the acceptability and willingness to use the service among PWID [22–26]. Research also suggests that intention to use SIS has been shown to predict actual use of SIS once such services are established [27]. Despite the need for widespread evidence-based harm reduction services in remote settings where drug use is a major concern [28, 29], a majority of research remains focused on PWID in urban settings. To-date, SIS feasibility research has been conducted in mid- to large-size North American urban centres [22–26], and as such, little is known about the acceptability and design preferences of SIS among PWID in remote settings.

**Fig. 1:**
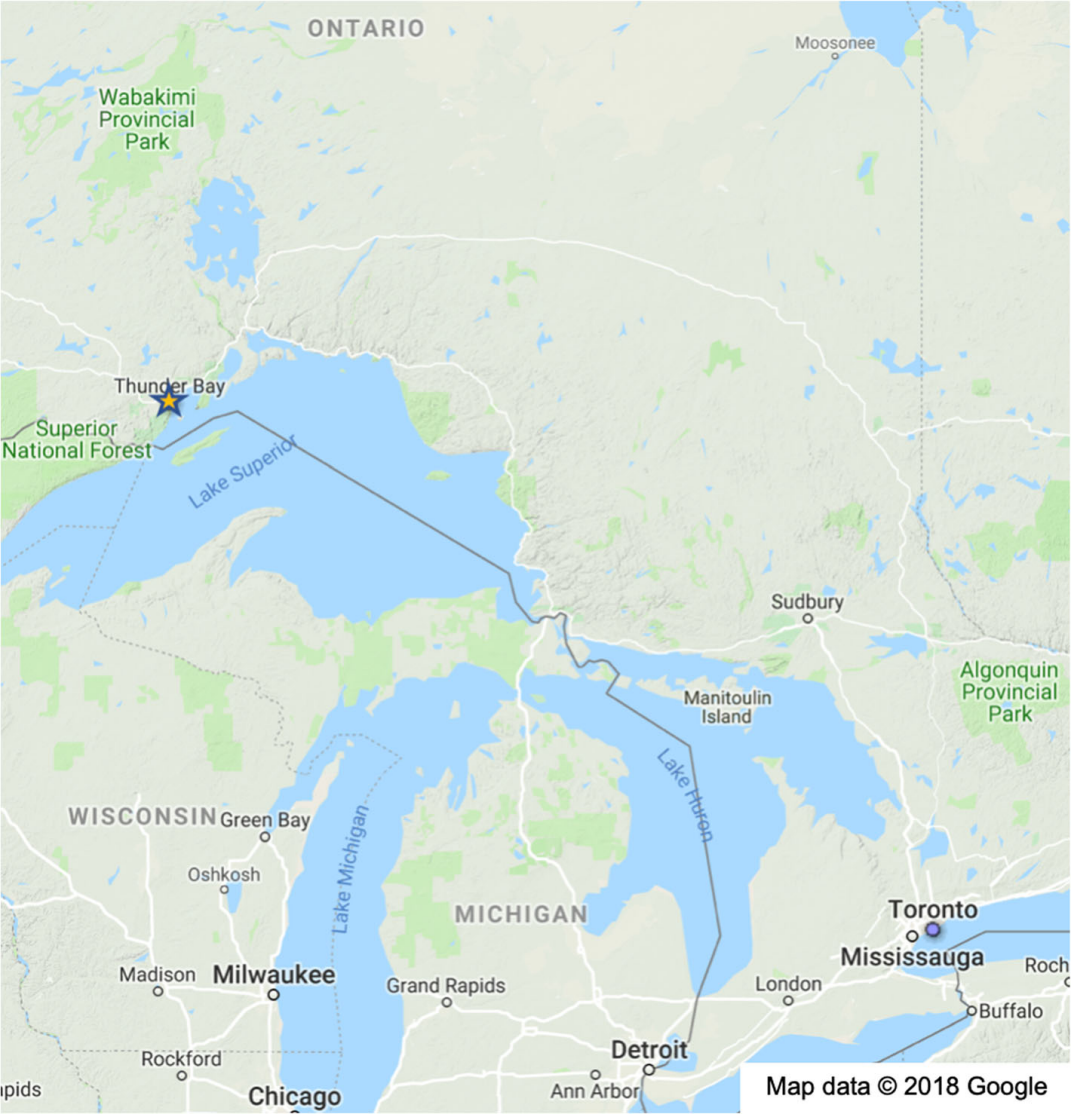
Location of Thunder Bay, Ontario. *Image attribution: Map data© 2018 Google*

Thunder Bay is a mid-sized city situated in the outlying and expansive region of Northwestern Ontario, Canada with a metropolitan population of 121,600 [30–32]. Located on the northern shore of Lake Superior, it is the most populous municipality in all of Northwestern Ontario (see Fig. 1 for map of Thunder Bay’s location). Since Thunder Bay is remotely situated from other neighbouring towns and cities, the city acts as a regional hub for surrounding rural and remote communities [30]. The Thunder Bay District Health Unit (TBDHU) which includes the City of Thunder Bay, oversees a geographic area of approximately 230,000 km^2^ and 146,000 residents [33]. Within the City of Thunder Bay, health and social service coverage spans large geographic areas and is intended to be reached by residents living in suburban, rural, and remote communities.

Illustrated by the present opioid and overdose crises spanning North America, many mid-size and remote cities are contending with issues related to overdose and injection drug use similar to that of larger urban centres [34]. Nonetheless, some evidence indicates that smaller and mid-size cities may experience different barriers to SIS implementation compared to larger urban centres. Common barriers experienced by PWID living in smaller and more remote communities include restricted access to a coordinated system of harm reduction services and addiction treatment, and concerns related to a lack of confidentiality and privacy when accessing substance use related care [29, 35]. Dispersed population density spanning large geographic areas in remote settings may further contribute to difficulty accessing services as a result of long distances to travel and inconsistent access to transportation [29]. Furthermore, some social norms characteristic of smaller and remote communities may introduce barriers related to access and implementation of harm reduction services. Compared to larger urban centres, smaller and more remote communities may sometimes face stringent views of individualism, self-sufficiency and conservatism toward substance use [29, 36]. Resulting stigma and discrimination may lead to social isolation or individuals choosing not to disclose their drug use, therefore making it challenging for some PWID to seek care [36]. Despite feasibility study results from another mid-size Canadian setting that found contrasting evidence to past research that suggests smaller cities lack liberal perspectives toward harm reduction and injection drug use [24], it remains unclear how these concerns may relate to the implementation of SIS in a geographically outlying mid-size city servicing suburban, rural and remote communities.

While there is limited information available on injection drug use in Thunder Bay, existing data suggests that the municipality has a disproportionately large population of PWID that is contending with a range of drug-related harms [37]. A 2013 community needs assessment revealed that the rate of non-prescription opioid use in Thunder Bay was higher than the provincial average (3.0% versus 1.7%) [38]. A 2018 surveillance report illustrated that between 2012 and 2016, the rate of emergency department visits for opioid overdose in the Thunder Bay region remained almost double that of the province of Ontario (ranging from 54.6–53.1 per 100,000 versus 23.5–31.7 per 100,000) [39]. With regards to injection drug use, a 2014 survey of PWID in Thunder Bay indicated high rates of needle and syringe borrowing and lending in the previous 6 months, at 19 and 21% respectively [40]. An enhanced surveillance reporting system among PWID across Canada revealed that between 2006-2007 and 2010–2012, the proportion of participants who injected opioids in the past 6 months in Thunder Bay substantially increased for almost all opioids, including morphine, oxycodone and fentanyl (an increase of 12.3, 35.4, and 7.1% respectively) [41]. Further, HCV rates in Thunder Bay are the second highest in the province of Ontario, and among those who reported injection drug use, 51% had lifetime exposure to HCV [40].

Thunder Bay supports one of the busiest needle syringe programs in the province [42], where clients can access sterile injection equipment such as needles, syringes, filters and cookers, are encouraged to return or properly dispose of needles or syringes after use, and are educated about the risks of using non-sterile equipment [43]. Nonetheless, concerns related to injection drug use in Thunder Bay persist [44]. While community services are in place in relation to housing programs and addiction treatment, they are not always accessible due to long wait times. Although Thunder Bay’s local drug strategy is calling for the further development of harm reduction approaches [45], there remains a lack of SIS feasibility research conducted in remote settings, and the potential role and acceptability of these services in Thunder Bay remains unknown. Therefore, we sought to characterize willingness to use SIS and expected frequency of SIS use among PWID in Thunder Bay, Canada and explored design and operational preferences among PWID.

## Methods

Data were derived from the Ontario Integrated Supervised Injection Services (OiSIS) Feasibility Study, a cross-sectional study of PWID Thunder Bay, Ontario, Canada [46, 47]. The study was supported by the Ontario HIV Treatment Network in partnership with the Canadian Institutes of Health Research Centre for REACH in HIV/AIDS and the Thunder Bay Drug Strategy, a local coalition of more than 30 partner agencies and people with lived experience. It was also overseen by an Advisory Committee comprised of local healthcare and social service providers, and other key stakeholders.

Between June and October 2016, the research team worked with two trained peer research associates (PRAs) to recruit participants through city-wide peer outreach efforts, word of mouth, and recruitment flyers posted at local health and social service agencies. Eligibility criteria included being 18 or older and having injected drugs in the last 6 months. Potential participants were invited for an appointment or drop-in interview at one of three community sites. PRAs administered a quantitative survey programmed on electronic tablets to study participants. The survey took approximately 45 min to complete and was adapted from previous SIS feasibility studies [26]. Information elicited from the questionnaire included socio-demographics, social-structural exposures, drug-using behaviours and patterns, access to health services, willingness to use and operational preferences for SIS. All participants received a $25 honorarium and provided written informed consent. This study was approved by the University of Toronto and the University of British Columbia’s research ethics boards.

### Measures and outcomes

Our primary outcomes of interest were willingness to use SIS and expected frequency of SIS use. Study participants were first asked “Have you heard of supervised injection services (SISs)?” with response options that included yes and no. If participants responded no, they were read the following script and description of SIS: “For this interview, we want to use the same definition of SISs, to make sure that we’re talking about the same type of place. A supervised injecting service is a legally operated indoor facility where people come to inject their own drugs under the supervision of medically trained workers. People can inject there under safe and sterile conditions and have access to all sterile injecting equipment (cotton, cooker, water, etc.) and receive basic medical care and/or be referred to appropriate health or social services.”

For willingness to use, response options were dichotomized into yes (i.e. those willing to use SIS) and maybe/no (i.e. those who may be willing or not willing to use SIS). For expected frequency of SIS use, study participants were asked “If a SIS was established in a location convenient to you, how often would you use it?”. Response options included: always (100% of injections), usually (over 75% of injections), sometimes (between 25 and 75% of injections), occasionally (less than 25% of injections), and never and were subsequently dichotomized into always/usually and sometime/occasionally (i.e. “high” and “low” expected frequency of SIS use, respectively).

A range of socio-demographic variables were considered for this analysis, including gender (female versus male), age (in years), ethnicity (white versus other, defined as Black, First Nations, Metis, Inuit, Francophone, South Asian, East Asian, Arab/West Asian, Latin American/Central American/South American) and engagement in sex work in the past 6 months (yes versus no). Housing status was also considered. Given the number of housing response options available, to increase interpretability, a binary variable was created (homeless or unstably housed [including living in a place where people gather to use drugs, hospital, rented hotel/motel room, no fixed address, on the street, prison/jail/detention centre, rehab, rooming or boarding house, shelter or welfare residence, medical hostel, or transitional housing] versus living alone, with a partner, or with family/friends in a house or apartment). Drug use behaviours and patterns in past 6 months were considered and included: any public injecting (yes versus no), any injecting alone (yes versus no), any help needed during injecting (yes versus no), any syringe sharing (categorized as borrowing or loaning; yes versus no), daily opioid injecting, daily cocaine injecting, daily rock cocaine injecting (all defined as daily versus less than daily or never) and any Wellbutrin, Ritalin or Biphentin injecting (yes versus no). Given polysubstance use is common among PWID [48], frequency of use for most drugs were defined as daily versus less than daily or never to get a better sense of the primary substance used by an individual, and to distinguish between drug use intensities, both of which are important considerations in relation to willingness to use SIS. We also assessed lifetime history of drug overdose (yes versus no) and lifetime history of drug treatment (including past use of one or a combination of detox programs, opioid substitution therapy, addiction case management, drug court, residential drug treatment, and outpatient counselling; yes versus no).

Finally, data were obtained on design and operational preferences and included preferred set-up for injecting space, willingness to use an integrated service (i.e. providing SIS alongside other health services, including but not limited to basic medical care, access to sterile needles and injecting equipment, harm reduction education, and HIV/HCV testing), willingness to walk or bus, desired hours of operation, involvement of PWID in service delivery, and important amenities to provide alongside SIS (See Table 3 for a range of questions asked).

### Data analysis

Using descriptive statistics stratified by our primary outcomes of interest, we reported proportions for categorical variables and the median (and interquartile range) for age as a continuous variable. To model socio-demographic and drug using behaviours associated with the outcomes of interest, willingness to use SIS and expected frequency of SIS use, we used logistic regression. All variables were entered into a full multivariable logistic model to adjust for suspected and potential confounders. In a backwards, step-wise manner, we dropped the least significant variable from the full model unless dropping the variable changed the statistical significance of other variables or resulted in potential changes in the point estimate. In an iterative process, reduced models were refit until all retained variables were either significant (*p* < 0.05) or identified as potential confounders. We also reported design and operational preferences and important amenities for using SIS stratified by gender. All analyses were conducted in SAS 9.4 [49].

## Results

Of 200 participants who provided complete data on willingness to use SIS (Table 1), the median age was 35 years (IQR: 28–43), 43% were female and 63% had previously heard of SIS. Sixty-nine percent (*n* = 137) reported willingness to use SIS. In bivariable analyses, those who expressed willingness to use were more likely to be unstably housed (Odds Ratio (OR): 2.10; 95% confidence interval (CI): 1.08–3.74) and more likely to report public injecting (OR: 4.61; 95% CI: 2.44–8.70), daily cocaine injecting (OR: 2.78; 95% CI: 1.09–7.05) and involvement in sex work (OR: 5.61; 95% CI: 1.64–19.15). Wide CIs for the estimate of sex work may be explained by low cell counts, particularly among those not willing or maybe willing to use SIS and engaging in sex work. In multivariable analyses, any public injecting in the last 6 months (Adjusted Odds Ratio (AOR): 4.15; 95% CI: 2.08–8.29) remained positively associated with willingness to use SIS.

**Table 1:** Socio-demographic, drug use characteristics and treatment history characteristics associated with willingness to use SIS (*n* = 200)

Characteristic	Total sample (*n* = 200)	Willingness to use SIS		Unadjusted OR (95% CI)	Adjusted OR (95% CI)
		Yes (*n* = 137) n (%)	No or Maybe (n = 63) n (%)		
Age, yr					
Median (IQR)	35 (28–43)	34 (27–43)	37 (28–46)	0.99 (0.96–1.02)	1.02 (0.98–1.05)
Gender					
Male	114 (57.0)	78 (68.4)	36 (31.5)	0.99 (0.54–1.81)	0.94 (0.47–1.85)
Female	86 (43.0)	59 (68.6)	27 (31.4)		
Ethnicity					
White	53 (26.5)	31 (58.5)	22 (41.5)	1.84 (0.95–3.53)	1.77 (0.81–3.87)
Other	147 (73.5%)	106 (72.1)	41 (27.9)		
Housing					
Unstable/Homeless	133 (66.5)	98 (73.7)	35 (26.3)	2.10* (1.08–3.74)	1.54 (0.75–3.16)
Stable	67 (33.5)	39 (58.2)	28 (41.8)		
Sex work					
Yes	33 (16.5)	30 (90.9)	3 (9.1)	5.61* (1.64–19.15)	-
No	167 (83.5)	107 (64.1)	60 (35.9)		
Any public injecting^a^					
Yes	128 (64.0)	103 (80.5)	25 (19.5)	4.61* (2.44–8.70)	4.15* (2.08–8.29)
No	72 (36.0)	34 (47.2)	38 (52.8)		
Any injecting alone^a^					
Yes	155 (78.0)	111 (71.6)	44 (28.4)	1.75 (0.87–3.50)	-
No	44 (22.0)	26 (59.1)	18 (40.9)		
Any help injecting^a^					
Yes	76 (38.0)	54 (71.1)	22 (28.9)	1.21 (0.65–2.25)	-
No	124 (62.0)	83 (66.9)	41 (33.1)		
Syringe sharing^a^					
Yes	23 (11.8)	14 (60.9)	9 (39.1)	0.71 (0.29–1.75)	-
No	172 (88.2)	118 (68.6)	54 (31.4)		
Daily opioid^b^ injecting^a^					
Yes	45 (22.5)	33 (73.3)	12 (26.7)	1.35 (0.64–2.83)	-
No	155 (77.5)	104 (67.1)	51 (32.7)		
Daily cocaine injecting^a^					
Yes	37 (18.5)	31 (83.8)	6 (16.2)	2.78* (1.09–7.05)	1.97 (0.73–5.31)
No	163 (81.5)	106 (65.0)	57 (35.0)		
Daily crack/rock cocaine injecting^a^					
Yes	21 (10.5)	17 (80.9)	4 (19.0)	2.09 (0.67–6.49)	-
No	179 (89.5)	120 (67.0)	59 (32.9)		
Any Wellbutrin injecting^a^					
Yes	67 (33.5)	51 (76.1)	16 (23.9)	1.74 (0.90–3.39)	
No	133 (66.5)	86 (64.7)	47 (35.3)		-
Any Ritalin or Biphentin injecting^a^					
Yes	75 (37.5)	56 (74.7)	19 (25.3)	1.60 (0.85–3.02)	-
No	125 (62.5)	81 (64.8)	44 (35.2)		
Ever OD					
Yes	77 (38.5)	57 (74.0)	20 (26.0)	1.53 (0.82–2.88)	-
No	123 (61.5)	80 (65.0)	43 (35.0)		
Drug treatment history					
Yes	143 (71.5)	99 (69.2)	44 (30.8)	1.13 (0.58–2.17)	-
No	57 (28.5)	38 (66.7)	19 (33.3)		

^*^p < 0.05

^a^In the last 6 months

^b^Opioids include Heroin, Hydros (Dilaudid and Hydromorph Contin), Generic Oxycodone, Oxy Neo, Percocet and Fentanyl

Of the 137 who reported willingness to use SIS, 135 (99%) provided complete data on expected frequency of SIS use (Table 2). Among those willing to use, 87 (64%) said they would always/usually use SIS, whereas 48 (36%) said they would use it sometimes/occasionally. In bivariable analyses, higher expected frequency of use was positively associated with being female (OR: 2.37; 95% CI: 1.12–5.03) and reporting any injecting alone (OR: 2.63; 95% CI: 1.09–6.34) and daily opioid injecting (OR: 3.15; 95% CI: 1.20–8.30). In multivariable analyses, being female (AOR: 2.44; 95% CI: 1.06–5.65) and any injecting alone (AOR: 2.59; 95% CI: 1.02–6.58) remained positively associated with higher expected frequency of use.

**Table 2:** Socio-demographic, drug use characteristics and treatment history characteristics associated with expected frequency of use among PWID willing to use SIS (*n* = 135)

Characteristic	Willing to Use (*n* = 135)	Expected frequency of use		Unadjusted OR (95% CI)	Adjusted OR (95% CI)
		Always/ Usually (*n* = 87) n (%)	Sometimes/ Occasionally (*n* = 48) n (%)		
Age, yr					
Median (IQR)	34 (27–43)	32 (25–41)	35 (20–48)	0.97 (0.94–1.01)	0.99 (0.94–1.02)
Gender					
Female	57 (42.2)	43 (75.4)	14 (24.6)	2.37* (1.12–5.03)	2.44* (1.06–5.65)
Male	78 (57.8)	44 (56.4)	34 (43.6)		
Ethnicity					
White	31 (23.0)	18 (58.1)	13 (41.9)	1.42 (0.63–3.24)	1.08 (0.40–2.95)
Other	104 (77.0)	69 (66.3)	35 (33.7)		
Housing					
Unstable/Homeless	98 (72.6)	64 (65.3)	34 (34.7)	1.15 (0.52–2.51)	0.79 (0.31–1.97)
Stable	37 (27.4)	23 (62.2)	14 (37.8)		
Sex work					
Yes	30 (22.2)	21 (70.0)	9 (30.0)	1.38 (0.57–3.31)	-
No	105 (77.8)	66 (62.9)	39 (37.1)		
Any public injecting^a^					
Yes	103 (76.3)	71 (68.9)	32 (31.1)	2.22 (0.99–4.98)	1.95 (0.78–4.88)
No	32 (23.7)	16 (50.0)	16 (50.0)		
Any injecting alone^a^					
Yes	38 (28.1)	30 (78.9)	8 (21.1)	2.63* (1.09–6.34)	2.59* (1.02–6.58)
No	97 (71.9)	57 (58.8)	40 (41.2)		
Any help injecting^a^					
Yes	53 (39.3)	29 (54.7)	24 (45.3)	0.50 (0.24–1.03)	-
No	82 (60.7)	58 (70.7)	24 (29.3)		
Syringe sharing^a^					
Yes	13 (9.8)	11 (84.6)	2 (15.4)	3.33 (0.70–15.57)	-
No	120 (90.2)	75 (62.5)	45 (37.5)		
Daily opioid^b^ injecting^a^					
Yes	33 (24.4)	27 (81.8)	6 (18.2)	3.15* (1.20–8.30)	2.64 (0.94–7.36)
No	102 (75.6)	60 (58.8)	42 (41.2)		
Daily cocaine injecting^a^					
Yes	30 (22.2)	24 (80.0)	6 (20.0)	2.67 (1.00–7.08)	-
No	105 (77.8)	63 (60.0)	42 (40.0)		
Daily crack/rock cocaine injecting^a^					
Yes	16 (11.9)	13 (81.3)	3 (18.7)	2.64 (0.71–9.76)	-
No	119 (88.1)	74 (62.2)	45 (37.8)		
Any Wellbutrin injecting^a^					
Yes	50 (37.0)	32 (64.0)	18 (36.0)		-
No	85 (63.0)	55 (64.7)	30 (35.3)	0.97 (0.47–2.01)	
Any Ritalin or Biphentin injecting^a^					
Yes	56 (41.5)	39 (69.6)	17 (30.4)	1.48 (0.72–3.07)	-
No	79 (58.5)	48 (60.8)	31 (39.2)		
Ever OD					
Yes	57 (42.2)	41 (71.9)	16 (28.1)	1.78 (0.86–3.71)	-
No	78 (57.8)	46 (59.0)	32 (41.0)		
Drug treatment history					
Yes	97 (71.9)	64 (66.0)	33 (34.0)	1.27 (0.58–2.74)	-
No	38 (28.1)	23 (60.5)	15 (39.5)		

*p < 0.05

^a^In the last 6 months

^b^Opioids include Heroin, Hydros (Dilaudid and Hydromorph Contin), Generic Oxycodone, Oxy Neo, Percocet and Fentanyl

Table 3 presents design and operational preferences among those willing to use SIS, stratified by gender. Of those willing to use SIS, 76% preferred private cubicle set-up for an injecting space, 63% preferred daytime operating hours, and 52% believed PWID should be involved in daily SIS operations. No differences were reported by gender. Eighty-eight percent were willing to walk to access SIS, while 78% were willing to take a bus. Women were less likely to report willingness to walk or bus 20 min or more to access SIS compared to men. Top services considered to be important to provide alongside SIS included distribution of needle and sterile injecting equipment, preventing and responding to overdose, HIV/HCV testing, and access to other health services. No differences were reported by gender.

**Table 3:** Design preferences and important amenities identified for SIS among PWID willing to use SIS (*n* = 137)

Design feature	Total (*n* = 137) n (%)	Males (*n* = 78) n (%)	Females (*n* = 59) n (%)	
Willing to walk to SIS	121 (88.3)	74 (94.9)	47 (79.1)	**
Willing to walk 20 min or more during Summer months	86 (62.7)	59 (75.6)	27 (45.8)	***
Willing to walk 20 min or more during Winter months	43 (31.4)	32 (41.0)	11 (18.6)	**
Willing to take a bus to SIS	107 (78.1)	66 (84.6)	41 (69.5)	NS
Willing to take a bus for 20 min or more during Summer months	88 (64.2)	57 (73.0)	31 (52.5)	*
Willing to take a bus for 20 min or more during Winter months	87 (63.5)	57 (73.0)	30 (50.8)	*
Preferred set-up for injecting space				
Private cubicle	103 (75.7)	57 (74.0)	46 (77.9)	NS
An open plan with benches at one large table or counter	5 (3.7)	4 (5.2)	1 (1.7)	
An open plan with tables and chairs	9 (6.7)	4 (5.2)	5 (8.5)	
A combination of above	19 (13.9)	12 (15.6)	7 (11.9)	
Preferred operating hours				
Daytime	79 (63.2)	44 (62.9)	35 (63.6)	NS
Evening	34 (27.2)	17 (24.3)	17 (30.9)	
Overnight	12 (9.6)	9 (12.8)	3 (5.5)	
Involvement of PWID in SIS operation	71 (51.8)	36 (46.2)	35 (59.3)	NS
Important amenities identified for SIS				
Needle distribution	136 (99.3)	77 (98.7)	59 (100)	NS
Distribution of sterile injection equipment	135 (98.5)	76 (97.4)	59 (100)	NS
Preventing or responding to overdose	134 (97.8)	75 (96.2)	58 (98.3)	NS
Access to health services	132 (96.4)	76 (97.4)	56 (94.9)	NS
HIV/HCV testing	131 (95.6)	75 (96.2)	56 (94.9)	NS
Withdrawal Management	129 (94.2)	76 (96.4)	53 (89.8)	NS
Washrooms	128 (93.4)	74 (98.9)	54 (91.5)	NS
Nursing staff for medical care and supervised injection teaching	127 (92.7)	70 (89.7)	57 (96.6)	NS
Harm reduction education	126 (92.0)	71 (91.0)	55 (93.2)	NS
Referrals to drug treatment, rehab, and other services when ready to use	124 (90.5)	70 (89.7)	54 (91.5)	NS

*NS* not significant at *p* < 0.05 level

*p < 0.05

**p < 0.01

***p < 001

## Discussion

We found moderately high levels of willingness to use SIS among PWID in the remote and mid-sized city of Thunder Bay, Canada. Willingness to use SIS was positively associated with public injecting in the last 6 months. Among those who expressed willingness to use SIS, higher expected frequency of SIS use was associated with being female and reporting any injecting alone in the past 6 months. PWID also expressed design and operational preferences, in addition to identifying other health and harm reduction services to provide alongside SIS.

Several North American urban centres have undertaken SIS feasibility research to determine the acceptability and role of SIS within communities [22–26]. Overall, levels of willingness to use SIS among large North American cities have been found to be high, ranging from 75 to 92% [22, 23, 26]. As a part of the Ontario Integrated Supervised Injection Services (OiSIS) [46, 47] Feasibility Study, recently published findings from a mid-sized urban centre found high levels of willingness to use SIS among PWID at 86% [24]. At 69%, we found a somewhat lower level of willingness to use SIS among PWID in this setting.

One possible explanation for the lower rate of willingness to use SIS in this context is related to stigma associated with injection drug use [29]. Rural and remote communities often contend with less liberal attitudes towards certain behaviours, including injection drug use [29, 36]. PWID from these communities may also lack anonymity [36] when accessing services and may experience fear surrounding breaches in confidentiality by healthcare providers [29]. Indeed, previous research has indicated that stigma related to injection drug use, and the consequent fear of disclosing one’s drug using behaviour through accessing services may prevent PWID from seeking appropriate treatment and care, and lead to reluctance to use such services [29]. This may be further complicated by socio-economic risk factors, including homelessness, food insecurity and poverty [29, 50]. Past successful strategies to address stigma associated with injection drug use and accessing harm reduction services in settings like Thunder Bay, which may also be considered when implementing SIS, include public media and education campaigns on the local realities of drug use [51], in addition to involvement of all affected communities in the planning and implementation of SIS to ensure respect and cultural safety.

While SIS appears acceptable among most PWID surveyed in Thunder Bay, it is important to consider the unique challenges that face health and social service practitioners providing care to suburban, rural, and remote populations accessing care in Thunder Bay. A majority of services, including harm reduction and addiction treatment in remote communities often remain underfunded and underserviced, with geographically extensive catchment areas, resulting in inadequate coverage and networks of care [28, 29]. Gaps in care are further complicated by long distances to services, and inconsistent public transportation [29]. Such limitations should be considered in the implementation of SIS.

Similar to previous SIS feasibility research, we found that willingness to use SIS was associated with injecting in public and semi-public spaces [22–24]. Public injection is viewed as a public nuisance within communities as a result of improperly discarded injection-related litter [5]. The act also poses risk to individual health through rushed injection practices and inability to ensure safety, privacy, and hygiene, resulting in an increased risk of syringe sharing, transmission of blood-borne infections and overdose [52–55]. Past evaluations have found that SIS can reduce risk of HIV transmission, syringe sharing, and fatal overdose among PWID, while also improving public order by reducing discarded syringes and injection related litter [5]. In this way, SIS may be an effective approach to reducing both the health and social harms associated with public injecting.

Among those who are willing to use SIS, we found that those who injected alone, and women reported higher expected frequency of SIS use. The harms associated with injecting alone have been well established in previous work. Individuals who inject alone are at elevated risk for fatal overdose [56–58] and have also been shown to be less knowledgeable about blood-borne infections, and less likely to attend harm reduction programs and addiction treatment [58]. The high expected frequency of SIS use among those who inject alone presents a crucial opportunity to prevent fatal overdose, and provide education on harm reduction and safe injection practices to this particularly vulnerable group. This finding is timely, and of significant importance given the present opioid and overdose epidemics affecting communities across North America [34].

Gender differences between men and women with respect to substance use, risk dynamics, and barriers to accessing addiction treatment have been well established [59, 60]. Compared to men who use drugs, women who use drugs experience increased threats of violence and greater stigma related to drug use, resulting in fear and shame and reluctance to seek appropriate care [61–63]. However, past research indicates that SIS can create a safe space for women who use drugs from the threats of violence in local drug scenes [64]. The role of harm reduction services, including SIS may be particularly important for women in Thunder Bay, given income security, limited access to childcare, and safe and affordable housing have been identified as major concerns for women who use substances in this setting [65]. Given that we found women were more likely to use SIS at a higher expected frequency than men, SIS may provide a unique opportunity to engage with women. Tailored strategies for women who use drugs that may be considered in this setting include a women’s only SIS, women’s specific drop-in hours, women-centered health and social services, and case management [59, 66, 67].

Among PWID willing to use SIS, a high proportion was willing to walk (88%) or take a bus (78%) to access SIS. Participants were more willing to walk longer distances (more than 20 min) in summer months compared to winter months (63% vs. 31%). There was, however, less difference between summer and winter months in terms of time participants were willing to take the bus (64% vs. 63%). These findings are somewhat in contrast to past SIS feasibility work in a large urban Canadian centre, where 36% of PWID were willing to travel more than 20 min to use SIS [22], and more consistent with feasibility findings in other mid-size settings where 60 and 40% of PWID were respectively willing to walk to the service in summer and winter months [24]. These findings suggest that in contrast to PWID in large urban centres, PWID in small to mid-sized cities may be willing to travel greater distances to access harm reduction services. Of note, we also found that women were overall less willing to walk or bus to services, and less willing to travel longer distances compared to men. Given that women expressed greater expected frequency of SIS use, gender differences in willingness to travel, and barriers to transportation should be taken into consideration to maximize uptake of the service. While this study did not look at the acceptability of mobile services, complementing a fixed-site SIS with mobile services may be considered as a suitable alternative to ensure coverage of geographically dispersed PWID in the Thunder Bay region [68]. However, a previous analysis found mobile SIS to be less cost-effective than fixed SIS sites [69].

Regarding design and operational preferences, a majority of participants preferred daytime hours of operation and private cubicles for injecting space set-up, while over half believed PWID should be involved in the day-to-day operations of service delivery. Involvement of peers in the delivery of harm reduction services may offer a unique opportunity to engage this typically hard-to-reach population, while also conferring benefits for the peers themselves [70]. Finally, in addition to naming a number of other harm reduction services to provide alongside SIS (i.e. distribution of sterile needles and injecting equipment and harm reduction education), other amenities deemed important include access to basic medical care, withdrawal management and the availability of washrooms. A similar combination of amenities, in addition to access to nursing staff, peer support, HIV/HCV testing, and referrals to drug treatment and services, were also identified in past feasibility studies conducted in the Canadian context [22, 24], highlighting PWIDs’ perspective of SIS as an integrated hub for health and wellbeing, beyond just a place to inject drugs.

This study has limitations. First, it relied on data from a non-randomized sample PWID and therefore may not be representative of all PWID in Thunder Bay. Similar to other studies with PWID, participants were recruited through diverse methods, including peer outreach efforts, word-of-mouth, and recruitment materials through local health and service organizations and therefore, our sample may be more likely to represent PWID willing to engage with health and harm reduction services, such as SIS. With the assistance of PRAs, efforts were made to recruit participants from a range of settings. However, this may have led to some groups or networks of individuals being over-represented in our sample. Second, having relied on self-reported responses obtained by PRAs, the data may have been subject to reporting biases including social desirability and recall bias. Nonetheless, past research suggests that self-reported responses from PWID are valid and reliable [71].

## Conclusions

In summary, we found moderately high levels of willingness to use SIS among PWID in Thunder Bay, Canada. Willingness to use SIS was positively associated with public injecting and higher expected frequency of use was associated with being female and injecting alone. Findings of the present study highlight the potential of SIS in addressing the harms associated with injection drug use in this remote and mid-sized setting, particularly among those who are most vulnerable. If implemented in Thunder Bay, to optimize the uptake of services, program planners and policy-makers should take into consideration the preferences and characteristics of local PWID, while also recognizing the unique challenges faced by PWID and harm reduction services in locations that serve the diverse needs of suburban, rural, and remote communities.

## Abbreviations

AOR: Adjusted odds ratio
CI: Confidence interval
HCV: Hepatitis C virus
HIV: Human immunodeficiency virus
OiSIS: Ontario Integrated Supervised Injection Services
OR: Odds ratio
PRA: Peer research associate
PWID: People who inject drugs
SIS: Supervised injection services
TBDHU: Thunder Bay District Health Unit
